# Peri-implant evaluation of osseointegrated implants subjected to orthodontic forces: results after three years of functional loading

**DOI:** 10.1590/2177-6709.21.2.073-080.oar

**Published:** 2016

**Authors:** Bruna de Rezende Marins, Suy Ellen Pramiu, Mauro Carlos Agner Busato, Luiz Carlos Marchi, Adriane Yaeko Togashi

**Affiliations:** 1 Graduate student in Oral and Maxillofacial Surgery, Universidade Estadual do Oeste do Paraná (UNIOESTE), School of Dentistry, Cascavel, Paraná, Brazil.; 2 Undergraduate student, Universidade Estadual do Oeste do Paraná (UNIOESTE), School of Dentistry, Cascavel, Paraná, Brazil.; 3 Professor, Universidade Estadual do Oeste do Paraná (UNIOESTE), Department of Orthodontics, School of Dentistry, Cascavel, Paraná, Brazil.; 4 Professor of Periodontology and Oral Implantology, Universidade Estadual do Oeste do Paraná (UNIOESTE), Department of Implantology, School of Dentistry, Cascavel, Paraná, Brazil.

**Keywords:** Bones, Dental implants, Orthodontic appliances, Osseointegration

## Abstract

**Objective:**

The objective of this study was to clinically and radiographically assess the peri-implant conditions of implants used as orthodontic anchorage.

**Methods:**

Two groups were studied: 1) a test group in which osseointegrated implants were used as orthodontic anchorage, with the application of 200-cN force; and 2) a control group in which implants were not subjected to orthodontic force, but supported a screw-retained prosthesis. Clinical evaluations were performed three, six and nine months after prosthesis installation and 1- and 3-year follow-up examinations. Intraoral periapical radiographs were obtained 30 days after surgical implant placement, at the time of prosthesis installation, and one, two and three years thereafter. The results were compared by Kruskal-Wallis test.

**Results:**

There was no statistically significant difference in clinical probing depth (*p* = 0.1078) or mesial and distal crestal bone resorption (*p* = 0.1832) during the study period. After three years of follow-up, the mean probing depth was 2.21 mm for the control group and 2.39 mm for the test group. The implants of the control group showed a mean distance between the bone crest and implant shoulder of 2.39 mm, whereas the implants used as orthodontic anchorage showed a mean distance of 2.58 mm at the distal site.

**Conclusion:**

Results suggest that the use of stable intraoral orthodontic anchorage did not compromise the health of peri-implant tissues or the longevity of the implant.

## RESUMO

**Introdução:**

o objetivo do presente estudo foi avaliar, clínica e radiograficamente, as condições peri-implantares de implantes usados como ancoragem ortodôntica.

**Métodos:**

dois grupos foram estudados: 1) Grupo Teste - no qual os implantes osseointegráveis foram utilizados como ancoragem ortodôntica, com aplicação de uma força de 200cN; e 2) Grupo Controle - no qual os implantes não foram submetidos à ancoragem ortodôntica, apenas serviram de suporte para fixação de prótese implantossuportada. Avaliações clínicas foram realizadas aos 3, 6 e 9 meses após a instalação das próteses, e após 1 e 3 anos de acompanhamento. Radiografias periapicais intrabucais foram obtidas 30 dias após a colocação do implante, no momento da instalação da prótese e após 1, 2 e 3 anos de acompanhamento. Os resultados foram comparados pelo teste de Kruskal-Wallis.

**Resultados:**

não houve diferenças quanto à profundidade clínica de sondagem (*p* = 0,1078) e a reabsorção das cristas ósseas mesial e distal (*p* = 0,1832) durante o período avaliado. Após três anos de acompanhamento, a média de profundidade de sondagem foi de 2,21mm para o Grupo Controle e de 2,39mm para o Grupo Teste. Os implantes do Grupo Controle apresentaram distância média de 2.39mm entre a crista óssea e o ombro do implante, enquanto os implantes usados como ancoragem ortodôntica mostraram distância média de 2,58mm na distal.

**Conclusão:**

esses resultados sugerem que o uso de uma ancoragem intrabucal estável não compromete a saúde dos tecidos peri-implantares ou a longevidade do implante.

## INTRODUCTION

Osseointegrated titanium implants were initially used as abutment for prosthetic reconstruction in fully edentulous patients in order to increase masticatory function.^1^ ^,^ ^2^ ^,^ ^3^ The implants were later used extensively to replace missing teeth in partially edentulous patients, allowing for preservation of the remaining dental structures.^1^ ^,^ ^4^ ^,^ ^5^ ^,^ ^6^ Other indications have been proposed for osseointegrated implants, such as orthodontic or maxillofacial anchorage,^7^ ^-^ ^11^ since decayed or missing teeth can impair orthodontic treatment due to absence of appropriate dental anchorage for orthodontic movement. 

The advantage of osseointegrated implants for orthodontic anchorage is the absolute immobility of the implant, as the periodontal ligament is inexistent, allowing for the application of controlled orthodontic forces without bone resorption. This phenomenon is known as "absolute anchorage."^12^ Thus, the implant will first function as intraoral orthodontic anchorage and later as prosthetic anchorage, providing stability, biocompatibility and comfort.^13^

Since several studies have demonstrated that anchorage of orthodontic forces on implants seems to be a good alternative in partially edentulous patients who require orthodontic treatment,^7^ ^,^ ^14^ ^-^ ^18^ the present study proposed to assess the long-term peri-implant behavior of implants subjected to orthodontic anchorage. Thus, the objective of this study was to clinically and radiographically assess implants used as orthodontic anchorage, as well as the success rate of such implants over a period of three years after the installation of prostheses over them.

## MATERIAL AND METHODS

### Patient selection and study design

A prospective clinical study using titanium implants as orthodontic anchorage was conducted. The patients were recruited from the Undergraduate and Postgraduate clinics of the Department of Dental Implantology, School of Dentistry, Universidade Estadual do Oeste do Paraná (UNIOESTE), Brazil. The study was approved by the Ethics Committee of the same university (Process 301/2008-CEP) and were asked to sign a free informed consent form after receiving detailed information about the study.

After patient selection according to inclusion and exclusion criteria, the sample was randomly divided into two groups: 1) test group in which osseointegrated implants were used as orthodontic anchorage (n = 26 implants); and 2) control group in which the implants were only used as support for prostheses (n = 24 implants).

Criteria for inclusion in the study were: 18 years of age or older (mean patient age was 41 years, with a range of 35 to 56 years old); willingness to cooperate with the requirements of the study; no systemic health condition; good oral hygiene; good periodontal health; sufficient alveolar bone volume at the implant recipient site (width: ≥ 6 mm and height: ≥ 8 mm) exclusively for the study group; and type I-III bone quality. Exclusion criteria were: pregnancy or breast-feeding; smoking and use of alcohol or drugs; previous reconstruction at the implant recipient site; insufficient alveolar bone volume at the implant recipient site (width: < 6 and height: < 8 mm); presence of residual roots at the recipient site; type IV bone quality; keratinized mucosa < 2 mm at the implant recipient site; stomatological and periodontal diseases; and clinical signs of temporomandibular dysfunction and bruxism.

The implants were installed according to the number of missing teeth and bone availability in the posterior region of the mandible, which required prosthetic rehabilitation and dental movements.

### Surgical procedures

Titanium implants were placed under local anesthesia by a dental surgeon and within a single intervention. The surgical procedure consisted of an incision in the alveolar ridge crest for preservation of the keratinized mucosa. Subsequently, lingual and buccal mucoperiosteal flaps were carefully elevated from the top of the alveolar crest. The implants were placed supracrestally, according to the protocol of the system, and primary stability was always achieved. The mucoperiosteal flaps were repositioned for healing by first intention. After one week, the sutures were removed and postoperative control was performed. The patient was advised to properly clean the treated area.

The surgical phase of implant installation consisted of the use of self-tapping external hexagon titanium implants (Dentoflex Comércio e Indústria de Materiais Odontológicos, São Paulo, Brazil), installed according to Branemark's surgical protocol.^19^ Implants with a diameter of 3.75 or 4.0 mm and 8, 10 and 11.5 mm in length were used according to bone availability.

Patients were advised to avoid any trauma to the implant sites and to rinse the mouth with 0.12% chlorhexidine digluconate for at least one minute, twice a day, for one week. 

### Clinical sequence

Four months after implant placement, the period corresponding to osseointegration, reopening and placement of healing abutments were performed. Subsequently, the implant was transferred and the crowns were screwed in place with a torque of 45 Nm. Molds of each patient were taken and each case was planned by an implantodontist and two orthodontists participating in the study.

Occlusion of the provisional acrylic resin screw-retained restorations was established with contact in maximum intercuspation and no contact in excursive movements. In the test group, provisional screw-retained restorations received the following orthodontic accessories: TMA wire cantilever (0.018 x 0.025-in, Morelli, Sorocaba, Brazil) and NiTi spring (0.25 mm diameter - Morelli, Sorocaba, Brazil). The maximum force applied to the implants was 200 cN. Orthodontic force was used in order to correct minor dental movements, such as molar uprighting and incisor relationship, and to improve the occlusal relationship with the objective of obtaining prosthetic space for implant placement. Orthodontic treatment period varied between 9 and 12 months. At the end of orthodontic treatment, provisional restorations were replaced with definitive prostheses.

### Clinical evaluation

Clinical evaluations were performed three, six and nine months after prosthesis installation. The 1- and 3-year follow-up examinations included the following parameters: 1) modified plaque index (mPlI)^20^ for all implants; 2) modified bleeding index (mBlI)^20^ for all implants; 3) pocket probing depth (PPD): distance between the gingival margin and pocket depth in millimeters;^21^ ^,^ ^22^ and 4) keratinized mucosa width (KMW): distance between the keratinized gingival-mucosa junction and the free gingival margin in millimeters for implants. All measurements were performed at six aspects of each implant site by means of a Hu-Friedy PCP-UNC probe (Hu-Friedy, Chicago, IL, USA) .

### Radiographic evaluation

**Figure 1:**
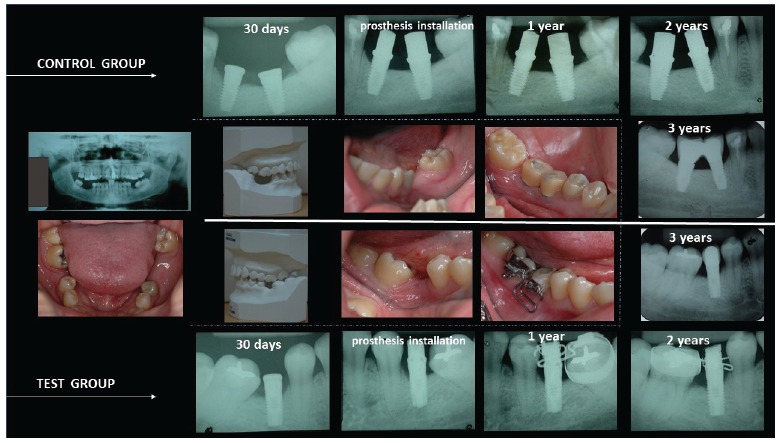
Intraoral periapical radiographs of the control and test groups before implant placement, 30 days after implant installation, at the time of prosthesis installation, and one, two and three years thereafter.

Intraoral periapical radiographs were obtained before implant placement and 30 days after surgery for implant placement at the time of prosthesis installation and one, two and three years thereafter (Fig 1). The paralleling technique was used. Radiographs were taken with the aid of a digital radiographic sensor (Kavo-Kerr), an individual acrylic positioning device, and an exposure time of 0.4 seconds. For the evaluation of changes in peri-implant crestal bone height, a single examiner measured the linear distance (in mm) from the implant shoulder to the most coronary part of the mesial and distal bone crest,^23^ using the Image Tool analysis program (UTHSCSA, Texas, USA).

Crestal bone measurements were made on the periapical radiographs obtained for the 8-mm, 10-mm and 11.5-mm implants. The length of the implant represents the reference to compensate for radiographic distortion. Subsequently, crestal bone measurements were obtained on the mesial and distal side for all implants at the pre-established intervals.^24^

### Follow-up and maintenance

Periodic visits were held for maintenance and reinforcement of oral hygiene instructions at 3-month intervals during the first year after prosthesis installation, and at 6-month intervals during the subsequent two years. 

Clinical parameters were evaluated at three, six and nine months and one and three years after prosthesis installation. Radiographic analysis was performed 30 days after surgery for implant placement at the time of prosthesis installation and one, two and three years thereafter. 

Success criteria established for the present study followed those of Karoussis et al;^21^ i.e., absence of mobility, absence of subjective complaints (pain, foreign body sensation, and/or paresthesia), no probing depth of 5 mm or more and positive modified sulcus bleeding index (mSBl), absence of continuous radiolucency around the implant, and an annual vertical bone loss not exceeding 0.2 mm after the first year since installation.

The results of the clinical parameters as well as bone crestal distance mesially and distally were compared by Kruskal-Wallis test. A *p*-value < 0.05 was considered to indicate statistical significance, and all calculations were performed by means of GraphPad InStat and GraphPad Prisma 5 software (GraphPad Software Inc, USA).

## RESULTS

Clinical, radiographic and peri-implant parameters showed that the biological response of gingival tissue and bone structure surrounding the implant subjected to orthodontic anchorage was similar to control. Peri-implant health was maintained for approximately one year of anchorage on the implant and over a period of three years of follow-up. 

**Figure 2:**
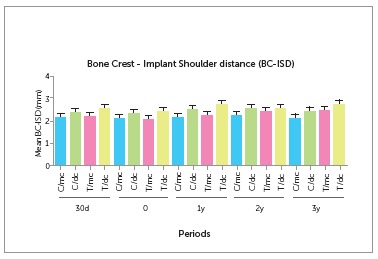
Comparison of the bone crest-implant shoulder distance of the implant during a period of 30 days after implant installation, at the time of prosthesis installation and after one, two and three years. Crestal bone measurements were obtained on the mesial (mc) and distal (dc) side for all implants, on groups Control (C) and Test (T), at the pre-established time points.

The mean bone crest/shoulder distance of the implant during a period of 30 days after implant installation, at the time of prosthesis installation, and one, two and three years thereafter revealed similar bone remodeling of the implant crests for the test and control groups, with no statistically significant difference between groups, since the time of implant placement, during the application of orthodontic force and throughout the study period (Fig 2 and Table 1). However, the mean bone crest/implant shoulder distance was 2.58 ± 1.19 mm on the distal surface for the test group and 2.39 ± 0.79 mm for the control group after three years of follow-up.

**Table 1:** Mean values of the bone crest/shoulder distance of the implant during a period of 30 days after implant installation, at the time of prosthesis installation and one, two and three years thereafter. Crestal bone measurements were obtained on the mesial (mc) and distal (dc) side for all implants at the pre-established time points.

Bone crest/shoulder distance of the implant (mm) mean ±SD											
Group	Periods										p
	30 days		0		1 year		2 years		3 years		
	mc	dc	mc	dc	mc	dc	mc	dc	mc	dc	
Control	2,13 (0.72)	2.33 (0.79)	2.09 (0.74)	2.27 (0.84)	2.15 (0.52)	2.47 (0.78)	2.22 (0.64)	2.53 (0.71)	2.14 (0.63)	2.39 (0.79)	0.1832 (NS)
Test	2.17 (0.74)	2.45 (1.02)	2.08 (0.59)	2.28 (1.03)	2.22 (0.68)	2.06 (0.96)	2.32 (1.02)	2.41 (1.11)	2.36 (0,92)	2.58 (1.19)	

Data are expressed as mean (SD). Kruskal-Wallis test, * *p* < 0.05; NS = non significant (*p* > 0.05). Crestal bone measurements: mesial (mc) and distal (dc) sides.

**Figure 3:**
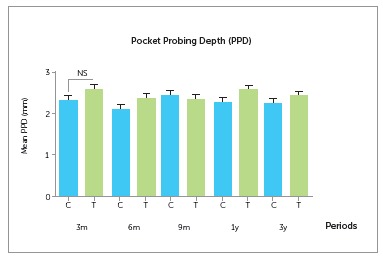
Comparison of pocket probing depth (PPD) measurements of implant at 3, 6 and 9 months and at 1 and 3 years after prosthesis installation, on Control (C) and Test (T) groups.

There was no significant difference in pocket probing depth between groups throughout the study period (Fig 3 and Table 2). The mean probing depth was 2.57 ± 0,40 mm and 2.39 ± 0.45 mm three months and three years after prosthesis installation, respectively, for implants of the test group, and 2.30 ± 0.54 mm and 2.21 ± 0.47 mm for the control group, showing that mean probing depth was unchanged throughout the study period. 

**Table 2:** Comparison of pocket probing depth (PPD) measurements of the implant at three, six and nine months and at one and three years after prosthesis installation.

Pocket probing depth (mm)						
Group	Periods					p
	3 m	6 m	9 m	1 y	3 y	
Control	2.30 (0.54)	2.10 (0.41)	2.39 (0.56)	2.25 (0.27)	2.21 (0.47)	*p* = 0.1078 (NS)
Test	2.57 (0.40)	2.39 (0.29)	2.24 (0.78)	2.51 (0.64)	2.39 (0.45)	

Data are expressed as mean (SD); Kruskal-Wallis test, *: *p* < 0.05; NS = non significant (*p* > 0.05).

**Figure 4:**
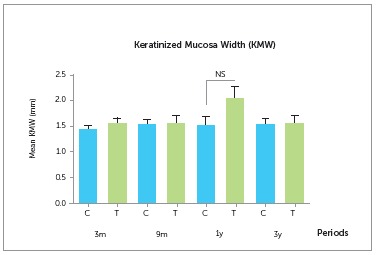
Comparison of keratinized mucosa width (KMW) measurements of the implant at 3 and 9 months and at 1 and 3 years after prosthesis installation, on Control (C) and Test (T) groups.

Keratinized mucosa width (KMW) did not differ significantly between groups during the study, with mean values of 1.43 ± 0.21 mm for the control group and 1.54 ± 0.40 mm for the test group, three months after prosthesis installation, and of 1.51 ± 0.47 mm and 1.56 ± 0.51 mm, respectively, after three years of follow-up (Fig 4 and Table 3). Thus, keratinized mucosa width remained stable and in sufficient quantity to protect the implant and the health of peri-implant tissue, providing better safety regarding the maintenance of peri-implant health.

**Table 3:** Comparison of keratinized mucosa width (KMW) measurements of the implant at three and nine months and at one and three years after prosthesis installation.

Keratinized mucosa width (mm)					
Group	Periods				p
	3 m	9 m	1 y	3 y	
Control	1.43 (0.21)	1.51 (0.34)	1.50 (0.64)	1.51 (0.47)	*p* = 0.1987 (NS)
Test	1.54 (0.40)	1.54 (0.55)	2.03 (0.85)	1.56 (0.51)	

Data are expressed as mean (SD); Kruskal-Wallis test, *: *p* < 0.05; NS = non significant (*p* > 0.05).

**Figure 5:**
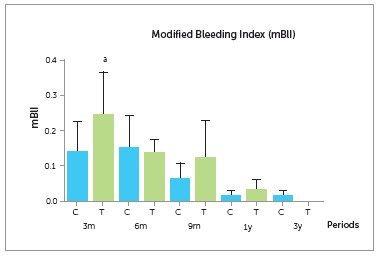
Comparison of the modified bleeding index (mBlI) for all implants at 3, 6 and 9 months and at 1 and 3 years after prosthesis installation.

The mean mBlI values did not differ significantly between groups, with both groups maintaining healthy peri-implant tissues throughout the three years of follow-up after prosthesis implantation (Fig 5 and Table 4). However, the test group revealed a trend towards an increase *(p* > 0.05) three and nine months after prosthesis installation.

**Table 4:** Comparison of the modified bleeding index (mBlI) for all implants at 3, 6 and 9 months and at 1 and 3 years after prosthesis installation.

Modified bleeding index						
Group	Periods					p
	3 m	6 m	9 m	1 y	3 y	
Control	0.13 (0.29)	0.15 (0.29)	0.06 (0.15)	0.01 (0.05)	0.01 (0.05)	0.017
Test	0.24 (0.40)	0.13 (0.12)	0.12 (0.35)	0.03 (0.10)	0.0 (0.0)	

Data are expressed as mean (SD). Kruskal-Wallis test: * = p < 0.05; superscript a = p < 0.05, for T9m group versus C6m and C3y groups; superscript b = p < 0.05, for T1y group versus C3m, T3m, C6m, T6m and C3y groups; NS = non significant: p > 0.05.

**Figure 6:**
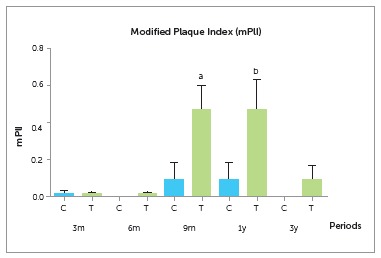
Comparison of the modified plaque index (mPlI) for all implants at 3, 6 and 9 months and at 1 and 3 years after prosthesis installation.

The mean mPlI values did not differ significantly between groups. By evaluating different periods of control and test groups, we observed an increase in the mean mPlI values in the test group at nine months and one year, compared to control at three, six months and three years, and test group at three and six months (Fig 6 and Table 5); thus, indicating that the test group presented significantly higher plaque formation on the peri-implant gingiva during the period orthodontic forces were applied to the implants.

**Table 5:** Comparison of the modified plaque index (mPlI) for all implants at 3, 6 and 9 months and at 1 and 3 years after prosthesis installation.

Modified plaque index						
Group	Periods					p
	3 m	6 m	9 m	1 y	3 y	
Control	0.01 (0.05)	0.0 (0.0)	0.09 (0.30)	0.09 (0.30)	0.0 (0.0)	p < 0.0001
Test	0.01 (0.04)	0.01 (0.04)	0.46 (0.49)^a^	0.46 (0.60)^b^	0.08 (0.27)	

Data are expressed as mean (SD); Kruskal-Wallis test: * = p < 0.05; superscript a = p < 0.05, for T3m group versus T3y group; NS = non significant (p > 0.05).

The rate of implant success was 100%, according to the criteria proposed by Karoussis et al.^21^ No implant showed radiographic changes in the bone-implant interface; no annual vertical bone loss exceeded 0.2 mm after the first year since installation, thereby indicating successful osseointegration of implants; and no implant mobility was observed. 

## DISCUSSION

Difficulty controlling anchorage is a significant aspect in Orthodontics. Standard anchorage devices, such as extraoral appliances and elastics, rely on patient's cooperation, which may compromise treatment results. The introduction of implants for orthodontic anchorage has decreased the need for patient's cooperation, compared to extraoral appliances, and has provided absolute anchorage biomechanics.^9^ ^,^ ^14^ ^,^ ^25^

In the present study, osseointegrated implants placed according to the method proposed by Branemark^19^ were clinically successful, fulfilling the proposed outcome criteria. In addition, the implants kept direct bone anchorage throughout the study period, in agreement with the results reported by Roberts et al^26^ and Higuchi and Slack.^8^ In the test group, it was possible to perform dental movement with an implant as anchorage, without any reciprocal action on the remaining teeth. The amount of peri-implant bone resorption of this group was similar to control.

Orthodontic forces on implants not only all the implants remained firm, but also maintained gingival relationships. This study provides evidence that orthodontic anchorage can have a favorable effect on marginal peri-implant gingival situation. In the test group, a slight increase (*p* > 0.05) was detected in keratinized mucosa width during orthodontic force application on implants, followed by a trend towards the reduction of such after three years of follow-up.

After the application of orthodontic force, there was clinical and radiographic peri-implant stability, as illustrated in Figures 1-6 and Tables 1-5. Results showed a 100% success rate for implants subjected to orthodontic forces of 200 cN; thus, indicating that, after orthodontic treatment, these implants can receive a fixed prosthesis replacing the missing teeth, in addition to improving patient's dental occlusion. Similarly, Cravero et al^7^ reported a 100% success rate and satisfactory occlusion with 93 implants placed in the maxilla and mandible.

In the present study, we observed that implants maintained direct bone anchorage throughout orthodontic treatment, in agreement with data reported by Roberts et al^26^ and Higuchi and Slack.^8^ Trisi et al^25^ also used implants for orthodontic anchorage in 41 adult patients. The implants were placed in different areas, all continued to be stable and were osseointegrated 12 months after prosthesis placement. These studies demonstrated that it was possible to perform small tooth movements without a reciprocal action, and that the dental occlusion of orthodontically treated patients was significantly improved. 

On the other hand, there have been no reports demonstrating the association between anchorage orthodontic and peri-implant conditions. Different time points of the test group showed an increase in the mean mPlI values after nine months and one year of prosthesis installation. This difference became more pronounced during the application of orthodontic forces. The results showed that the mean mPlI values in the test group with bonded orthodontic devices were higher in comparison to control group without orthodontic devices. The influence of impaired oral hygiene was considered; however, mPlI for the test group did not improve in spite of detailed oral hygiene instructions. The result of the mPlI reveals the difficulty performing oral hygiene for the test group during tooth movement. After completion of orthodontic treatment, the mean mPlI values became normal. Over a 3-year follow-up, peri-implant parameters were considered satisfactory in terms of gingival health. The reason for higher susceptibility to biological complications around implants may be discussed in the light of bacterial plaque accumulation in partially edentulous dentitions or the host response to the bacterial challenge. The microbiota associated with periodontitis and peri-implantitis has supported the concept that periodontal pathogens are important etiological factors of peri-implant infections.^20^ ^,^ ^27^ It is, therefore, obvious that the status of peri-implant health is of utmost importance for the longevity of implants installed.

According to Werbein and Merz^28^ and Pinho et al,^29^ intraosseous titanium implants yield the best results for orthodontic use; thus, reducing treatment time. 

Furthermore, osseointegrated implants have proved to resist displacement forces of 100 to 200 cN on all planes, and to function as a unit of orthodontic anchorage.^7^ ^,^ ^30^ After orthodontic treatment, implants also served as abutment for permanent fixed prostheses in edentulous areas, which could not have been properly carried out without implant anchorage.

## CONCLUSION

On the basis of the present results, we suggest that subjecting osseointegrated implants to orthodontic forces can be a safe technique for prosthetic rehabilitation and an alternative for the orthodontic treatment of partially edentulous patients, since there was no significant peri-implant bone loss after orthodontic activation. Additionally, there were no changes in peri-implant probing depth or in the gingiva, and no bleeding or presence of peri-implant plaque was observed during a 3-year follow-up after installation of implant-supported prosthesis.

## References

[1] Adell R, Lekholm U, Rockler B, Branemark PI (1981). A 15-year study of osseointegrated implants in the treatment of the
edentulous jaw. Int J Oral Surg.

[2] Carrión JP, Barbosa IR, López JP. (2009). Osseointegrated implants as orthodontic anchorage and restorative
abutments in the treatment of partially edentulous adult patients.. Int J Periodontics Restorative Dent..

[3] Jones SD, Jones FR. (1988). Tissue integrated implants for the partially edentulous
patient.. J Prosthet Dent..

[4] Lekholm U, Gunne J, Henry P, Higuchi K, Linden U, Bergstrom C (1999). Survival of the branemark implant in partially edentulous jaws a
10-year prospective multicenter study. Int J Oral Maxillofac.

[5] Albrektsson T, Dahl E, Enbom L, Engevall S, Engquist B, Eriksson AR (1988). Osseointegrated oral implants a Swedish multicenter study of 8139
consecutively inserted nobelpharma implants. J Periodontol.

[6] Oldman J, Lekholm U, Kholm U, Jemt T, Branemark PI, Thilander B (1988). Osseointegrated titanium implants a new approach in orthodontic
treatment. Eur J Orthod.

[7] Cravero RM, Ibañez JC (2008). Assessing double acid-etched implants submitted to orthodontic forces
and used as prosthetic anchorages in partially edentulous patients. Open Dent J.

[8] Higuchi KW, Slack JM (1991). The use of titanium fixtures for intraoral anchorage report of a
case. Int J Oral Maxillofac Implants.

[9] Block MS, Hoffman DR (1995). A new device for absolute anchorage for orthodontics. Am J Orthod Dentofacial Orthop.

[10] Goto M, Jin-Nouchi S, Ihara K, Katsuki T (2002). Longitudinal follow-up of osseointegrated implants in patients with
resected jaws. Int J Oral Maxillofac Implants.

[11] Roumanas ED, Freymiller EG, Chang TL, Aghaloo T, Beumer J (2002). Implant-retained prostheses for facial defects an up to 14-year
follow-up report on the survival rates of implants at UCLA. Int J Prosthodont.

[12] Liaw Yc, Kuang Sh, Chen Yw, Hung Kf, Tsai Hc, Kao Sy (2008). Multimodality treatment for rehabilitation of adult orthodontic
patient with complicated dental condition and jaw relation. J Chin Med Assoc.

[13] Cochran DL, Bosshardt DD, Grize L, Higginbottom FL, Jones AA, Jung RE (2009). Bone response to loaded implants with nonmatching implant abutment
diameters in the canine mandible.. J Periodontol..

[14] Thilander B, Odman J, Lekholm U (2001). Orthodontic aspects of the use of oral implants in adolescents a
10-year follow-up study. Eur J Orthod.

[15] Drago CJ (1999). Use of osseointegrated implants in adult orthodontic treatment a
clinical report. J Prosthet Dent.

[16] Willems G, Carels CE, Naert IE, Van Steenberghe D (1999). Interdisciplinary treatment planning for orthodontic-prosthetic
implant anchorage in a partially edentulous patient. Clin Oral Implants Res.

[17] Goodacre CJ, Brown DT, Roberts WE, Jeiroudi MT (1997). Prosthodontic considerations when using implants for orthodontic
anchorage. J Prosthet Dent.

[1] 8 Celenza F (2003). Implant-enhanced tooth movement indirect absolute
anchorage. Int J Periodontics Restorative Dent.

[19] Bränemark P-I (1977). An experimental and clinical study of osseointegrated in treatment of
the edentulous jaws Experience from a 10-year period. Scand J Plast Reconstr Surg.

[20] Mombelli A, Van Oosten MAC, Schurch E, Lang NP (1987). The microbiota associated with successful or failing osseointegrated
titanium implants. Oral Microbiol Immunol.

[21] Karoussis IK, Salvi GE, Heitz-Mayfield LJ, Brägger U, Hämmerle CH, Lang NP (2003). Long-termimplant prognosis in patients with and without a history of
chronic periodontitis a 10-year prospective cohort study of the ITIs Dental
Implant System. Clin Oral Implants Res.

[22] Karoussis IK, Müller S, Salvi GE, Heitz-Mayfield LJ, Brägger U, Lang NP (2004). Association between periodontal and peri-implant conditions a 10-year
prospective study. Clin Oral Implants Res.

[23] Smith DE, Zarb G (1989). Criteria for success of osseointegrated endosseous
implants. J Prosthet Dent.

[24] Weber HP, Buser D, Donath K, Fiorellini JP, Doppalapudi V, Paquette DW (1996). Comparison of healed tissues adjacent to submerged and non-submerged
unloaded titanium dental implants A histometric study in beagle
dogs. Clin Oral Implants Res.

[25] Trisi P, Rebaudi A (2002). Progressive bone adaptation of titanium implants during and after
orthodontic load in humans. Int J Periodontics Restorative Dent.

[26] Roberts WE, Engen DW, Schneider PM, Hohlt WF. (2004). Implant anchored orthodontics for partially edentulous malocclusions
in children and adults.. Am J Orthod Dentofacial Orthop..

[27] Leonhardt A, Adolfsson B, Lekholm U, Wikström M, Dahlén G. (1993). Longitudinal microbiological study on osseointegrated titanium
implants in partially edentulous patients.. Clin Oral Implants Res..

[28] Wehrbein H, Merz BR, Hammerle CH, Lang NP (1998). Bone-to-implant contact of orthodontic implants in humans subjected to
horizontal loading. Clin Oral Implants Res.

[29] Pinho T, Neves M, Alves C (2012). Multidisciplinary management including periodontics, orthodontics,
implants, and prosthetics for an adult. Am J Orthod Dentofacial Orthop.

[30] Palagi LM, Sabrosa CE, Gava EC, Baccetti T, Miguel JA. (2010). Long-term follow-up of dental single implants under immediate
orthodontic load.. Angle Orthod..

